# AKAR2-AKAP12 fusion protein *"biosenses*" dynamic phosphorylation and localization of a GPCR-based scaffold

**DOI:** 10.1186/1750-2187-5-3

**Published:** 2010-04-22

**Authors:** Jiangchuan Tao, Hsien-yu Wang, Craig C Malbon

**Affiliations:** 1 Department of Pharmacology, School of Medicine, Heath Sciences Center, SUNY/Stony Brook, Stony Brook, NY 11794-8651, USA; 2 Department of Physiology & Biophysics, School of Medicine, Heath Sciences Center, SUNY/Stony Brook, Stony Brook, NY 11794-8651, USA

## Abstract

**Background:**

The cAMP-dependent protein kinase A (PKA) plays a pivotal role in virtually all cells, there being a multitude of important target molecules that are substrates for PKA in cell signaling. The spatial-temporal dynamics of PKA activation in living cells has been made accessible by the development of clever biosensors that yield a FRET signal in response to the phosphorylation by PKA. AKAR2 is genetically encoded fluorescent probe that acts as a biosensor for PKA activation. AKAP12 is a scaffold that docks PKA, G-protein-coupled receptors, cell membrane negatively-charged phospholipids, and catalyzes receptor resensitization and recycling. In the current work, the AKAR2 biosensor was fused to the N-terminus of AKAP12 to evaluate its ability to function and report on dynamic phosphorylation of the AKAP12 scaffold.

**Results:**

AKAR2-AKAP12 can be expressed in mammalian cells, is fully functional, and reveals the spatial-temporal activation of AKAP12 undergoing phosphorylation by PKA in response to beta-adrenergic activation in human epidermoid carcinoma A431 cells.

**Conclusion:**

The dynamic phosphorylation of AKAP12 *"biosensed*" by AKAR2-AKAP12 reveals the scaffold in association with the cell membrane, undergoing rapid phosphorylation by PKA. The perinuclear, cytoplasmic accumulation of phosphorylated scaffold reflects the phosphorylated, PKA-activated form of AKAP12, which catalyzes the resensitization and recycling of desensitized, internalized G-protein-coupled receptors.

## Background

The discovery of a class of scaffold proteins that harbor a binding site for the regulatory subunits (*i.e*., RI/RII) of cyclic AMP-dependent protein kinase A (PKA, A-kinase) was seminal in our understanding of the roles of these A-Kinase Anchoring Proteins (AKAPs), in many facets of cellular signaling [1]. AKAPs not only dock PKA, but also can act as molecular "tool boxes" that capable of docking protein kinases other than PKA (including protein kinase C, PKC, and the Src-family tyrosine kinases), phosphoprotein phosphatases (such as protein phosphatase-2B), and adaptor molecules [2]. AKAPs participate dynamically in such large, macromolecular signaling complexes that can include not only protein kinases and phosphoprotein phosphatases, but also phosphodiesterases (PDE), adaptor molecules (like Grb2), ion channels, and members of the superfamily of G protein-coupled receptors (GPCR) [3]. Two AKAPs, AKAP12 (as known as AKAP250, gravin, and SSECKS) and AKAP5 associate with the beta-adrenergic receptors and have been the focus of intense research [2-6]. Human epidermoid carcinoma A431 cells express AKAP12 and a full complement of the prototypic GPCR, the beta_2_-adrenergic receptor [7-10]. A431 cells have been well-characterized with respect to PKA-based cell signaling [7-10] and were adopted for the current studies.

One of the major obstacles in understanding the precise functions of signaling molecules like AKAP scaffolds is the inability to ascertain the spatial-temporal dynamics of the molecules during activation of a signaling pathway [11]. Most approaches (e.g., immunohistochemical and imaging with autofluorescent-tagged molecules) provide signals from the *entire *cellular complement of a specific molecule, when what truly is sought is study of only the subset of these molecules that are actively involved in the signaling, which may represent a vanishing small percentage. The advent of powerful *"biosensors*" that can report on changes in function as a result of activation of a kinase (e.g., AKAR/AKAR2 reporting phosphorylation events catalyzed by PKA) provide a novel avenue with which to study of the spatial-temporal signaling of PKA [12]. AKAR2, employed in this study, is a biosensor for PKA phosphorylation that includes the cyan fluorescent protein (CFP), a consensus substrate sequence for PKA-catalyzed phosphorylation, a phosphoamino acid-binding sequence of the Forkhead Homology domain, and citrine, a less pH-sensitive variant of enhanced yellow fluorescent protein (YFP) [11,12]. Phosphorylation of the PKA consensus sequence of AKAR2 induces tight binding of the phosphoamino acid-binding domain provoking proximity of CFP and YFP, generating fluorescence resonance energy transfer (FRET) [11,12]. We have sought a hybrid approach that combines the power of the AKAR2 biosensor with a desire to measure not all of the events catalyzed by PKA, but only those confined intentionally to a single PKA substrate AKAP12 [13-15]. PKA phosphorylation of AKAP12 is central to the function of this scaffold, essential to the ability of AKAP12 to resensitize and recycle GPCRs in cells following the activation of adenyl cyclase, a rise in intracellular cyclic AMP, and the activation of PKA [14]. PKA-catalyzed phosphorylation also regulates other AKAP scaffolds that bind GPCRs [1,5,6,15]. Herein, we seek to probe the dynamic, spatial-temporal character of AKAP12 by creating a fusion protein of the biosensor AKAR2 with the AKAP12. We characterize a fusion construct that retains all the functional capability of the AKAP12, but can, by virtue of its AKAR2 moiety, report as a biosensor on the phosphorylation of AKAR2-AKAP12 in living cells.

## Results and Discussion

**Figure 1:**
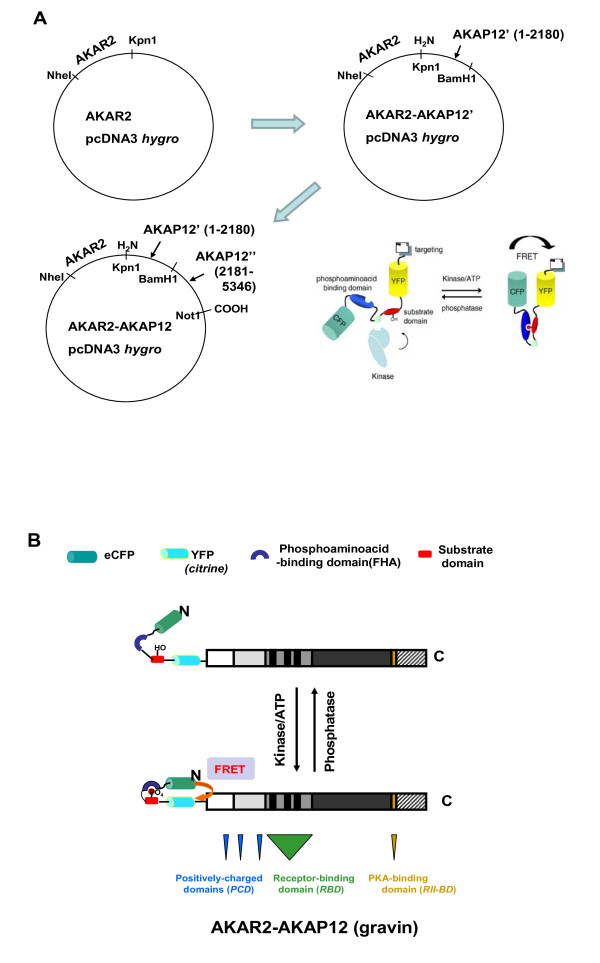
**Strategy for construction of plasmid encoding a targeting biosensor, AKAR2-AKAP12**. *Panel A*, AKAR2 (a generous gift of the laboratory of Dr. Roger Tsien (UCSD) was engineered to the N-terminal of AKAP12, in two steps. The AKAP12 N-terminal half of the sequence (nucleotides 1-2180) was inserted as a Kpn1-BamH1 fragment in to the AKAR2 pcDNA3 *hygro *plasmid (pAKAR2-AKAP12'), as detailed in the *Methods *section. The C-terminal remainder of the AKAP12 molecule (nucleotides 2181-5346, AKAP12") was inserted as a BamH1-Not1 fragment into the pAKAR2-AKAP12' plasmid to generate the final construct, AKAR2-AKAP12. The AKAR2 moiety is composed of an N-terminal CFP, the Forkhead phosphoamino acid-binding domain (blue oval), docking site and substrate domain (red oval) with specificity for PKA-catalyzed phosphorylation, and a C-terminal YFP (citrine variant) which is fused to the N-terminus of the AKAP scaffold protein (i.e., the "targeting" moiety for the biosensor), AKAP12. *Panel B*, the docking of the kinase and phosphorylating the substrate site lead to a strong interaction with the Forkhead phosphoaminoacid-binding domain. This phosphate group-FHA domain binding brings the CFP and YFP moieties into close proximity, enabling FRET signaling. Phosphoprotein phosphatase hydrolyzes the phosphophate group and relaxes the interaction between CFP and YFP, attenuating the capacity for FRET.

**Figure 2:**
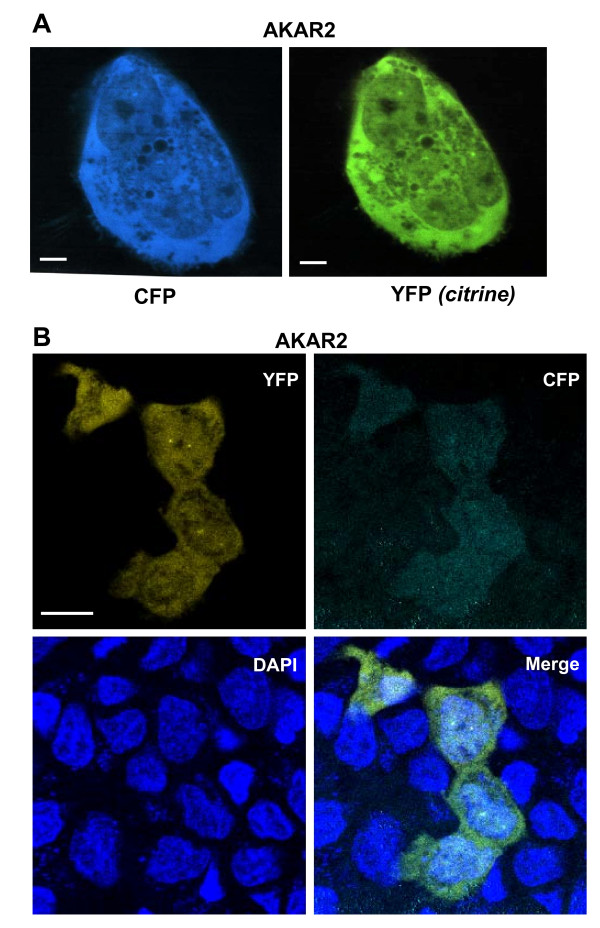
**Imaging of AKAR2 expressed in human epidermoid carcinoma A431 cells**. *Panel A*, images of AKAR2 biosensor when expressed in A431 cells. The fluorescence images were recorded at 473-495 nm for CFP, and, 527-591 nm for YFP (citrine). *Panel B*, AKAR2-based fluorescence images were recorded at 473-495 nm for CFP and 527-591 nm for YFP (citrine), in A431 cells in which the nuclei were stained with DAPI. The DAPI recordings were performed at 435-485 nm. The images shown are representative of a large array of images collected for this purpose.

The biosensor moiety for protein kinase A-dependent phosphorylation, AKAR2 [12], was fused to the N-terminus of AKAP12 in a simple two-step strategy (Fig. 1A). The positively-charged domains (PCDs) are localized in the N-terminus of AKAP12 [13]. The addition of the AKAR2 moiety to the N-terminus necessarily removes the N-myristoylation of the native protein. Earlier it was shown that in the presence of three PCDs, the loss of the N-myristoylation site does not alter the ability of AKAP12 to function [13]. Thereceptor-binding domain (RBD) for GPCRs is located close to the mid-point of AKAP12 [14] and the binding site for the RII subunit of protein kinase A is located in the C-terminal region of the molecule (Fig. 1B) [1-3]. We investigated the expression of the AKAR2 moiety alone, as a control, by transient transfection in human epidermoid carcinoma A431 cells (Fig. 2A). The A431 cells were imaged at 473-495 nm for CFP and 527-591 for YFP (in this case, the citrine variant). Excepting the nucleus, the distribution of AKAR2 expressed in A431 cells was uniform throughout cells, as made clear by the fluorescent signals from the CFP and YFP moieties of the AKAR2 molecule. Using the same approach to highlight the nucleus by DAPI staining of live cells, the distribution of AKAR2 throughout the cell, excluding the nucleus, can be highlighted in the "merge" panel (Fig. 2B).

**Figure 3:**
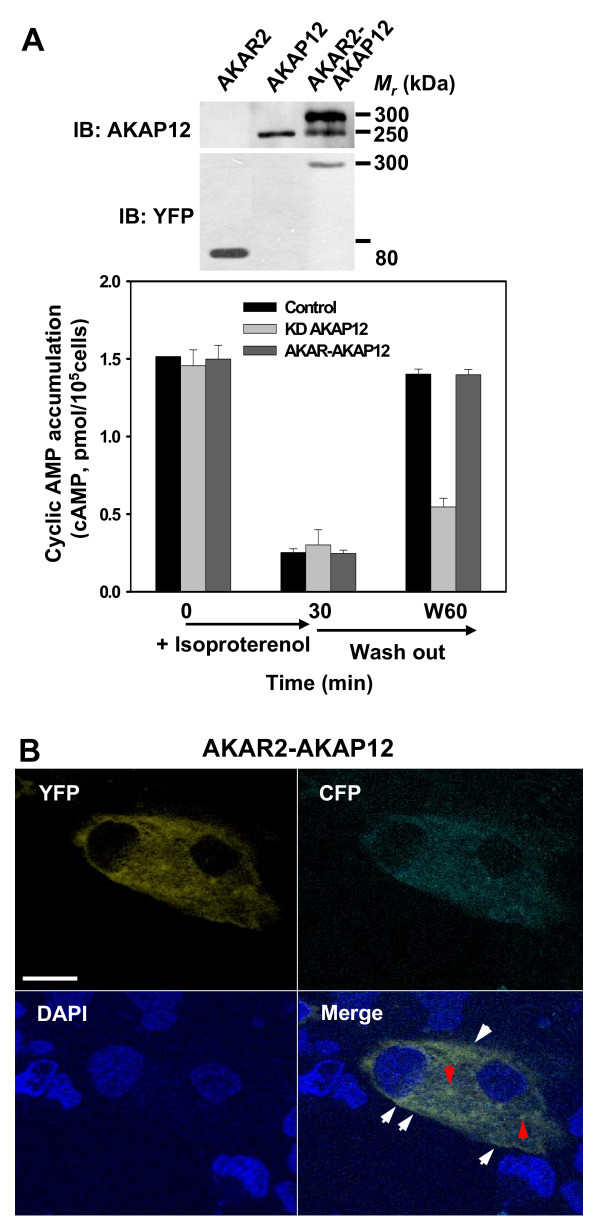
**Expression of AKAR2-AKAP12 fusion protein in A431 cells: *M*_*r*_, function, and imaging**. A431 cells were transfected with an expression vector plasmid harboring one of the following cDNAs: AKAR2; AKAP12; and AKAR2-AKAP12. *Panel A *(top), the expression of AKAR2, AKAP12, and AKAR2-AKAP12 was examined. The stained immunoblots and the approximate *M*_*r *_of each resolved band is indicated. *Panel A *(bottom), A431 cells were challenged with β-agonist (isoproterenol, 10 μm) for 5 min and the intracellular cAMP (pmol/10^5 ^cells) represents the normal response of naïve A431 cells (at time = "0"). Upon a 30 min challenge with isoproterenol, cells became desensitized to a second, subsequent challenge with the β-agonist (30). The agonist was then washed out and the cells were allowed to recover. The "resensitization" was measured in cells at 60 min following the washout (*W60*). This activation, desensitization, wash out and recovery were performed on A431 cells alone (Control), on AKAP12-deficient cells (*i.e*., KD AKAP12), and on AKAP12-deficient cells transiently expressing AKAR2-AKPA12. The results, displayed as mean values ± S.E., are of at least three separate experiments. *Panel B*, imaging of cellular distribution of AKAR2-AKAP12 in A431 cells. The fluorescence images were recorded at 473-495 nm for CFP and 527-591 nm for YFP. Nuclei stained with DAPI were recorded at 435-485 nm. The images shown are representative of a large array of images collected for this purpose.

The AKAR2-AKAP biosensor was constructed in pcDNA3 expression vector and A431 cells were transiently transfected (Fig. 3A). Expression of exogenous AKAP12, the AKAR2 biosensor itself, or the complete AKAR2-AKAP12 fusion biosensor protein was examined in whole-cell lysates of A431 cells transfected with one of the three plasmids. The lysates were subjected to SDS-PAGE, the resolved proteins transferred to blots, and the blots subjected to staining with primary antibodies against either AKAP12 or against the YFP moiety. The immunoblots reveal the expression of AKAR2 (*M*_*r *_= 80 kDa), of AKAP12 itself (*M*_*r *_= 250 kDa), and of the AKAR2-AKAP12 fusion biosensor (*M*_*r *_~300 kDa). It was important to determine if the fusion protein retained biological function. AKAP12 plays an essential role in the resensitization of beta_2_-adrenergic receptors that have undergone agonist-induced desensitization [1-3]. Treating cells with the beta-adrenergic agonist isoproterenol for 30 min ultimately desensitizes the treated cells, *i.e*., sharply attenuates the accumulation of intracellular cyclic AMP in response to a second stimulation with beta-adrenergic agonist (Fig. 3A, bottom panel). A wash-out of agonist followed by a 60-min recovery period (W60) leads to full functional recovery in native A431 cells, but not in cells treated with morpholinos antisense to the 5'-untranslated region of AKAP12 (KD AKAP12), *i.e*., morpholinos designed to suppress expression of endogenous AKAP12 only [13]. Morpholinos antisense for AKAP12 are used routinely to suppress the endogenous AKAP12; typically, the reduction is more than 90% [13, and data not shown]. Expression of the AKAR2-AKAP12 fusion biosensor restores the ability of the cells to fully recover from desensitization, rescuing the ability of the beta_2_-adrenergic receptors of AKAP12-deficient cells to function. Thus, a fusion protein of AKAR2 N-terminal to AKAP12 is readily expressed and displays function.

In the absence of any stimulation with a beta-adrenergic agonist, A431 cells expressing AKAR2-AKAP12 display a pattern of distribution very similar to that observed earlier for eGFP-tagged AKAP12 in intact cells [13] and much earlier for immunohistochemical staining of AKAP12 in fixed cells [15]. By imaging the fluorochrome reporters, AKAR2-AKAP12 can be localized in association with the cell membrane (white arrows) as well as in large, punctiform clusters found in the perinuclear compartment of the cytosol (red arrows, Fig 3B). The enhanced localization of the scaffold biosensor at the cell membrane reflects the presence of three PCDs in the N-terminal domain of AKAP12 [13]. Both AKAP5 and AKAP12 share the presence of three PCDs and both scaffolds are found at higher relative concentrations in close proximity to the negatively-charged cell membrane [16].

**Figure 4:**
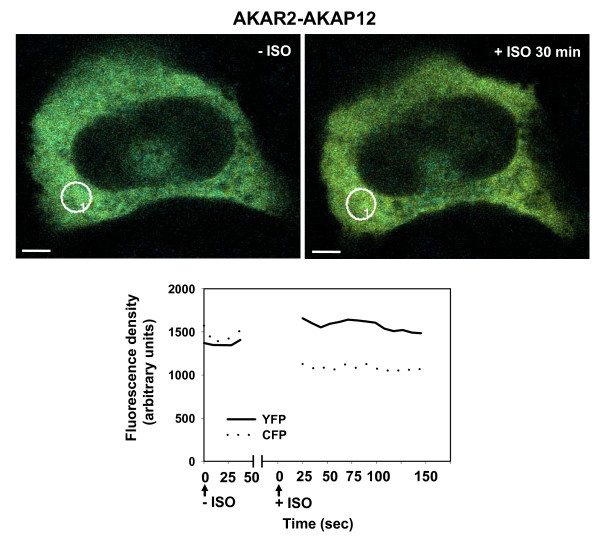
**AKAR2-AKAP12 fusion protein biosensor: FRET signaling in response to beta-adrenergic stimulation**. Stably transfected A431 clones expressing AKAR-AKAP12 were serum starved over night and then stimulated with beta-adrenergic agonist (isoproterenol, 10 mM) for 30 min. Image and fluorescence density were recorded for CFP and YFP in the absence and following the 30 min challenge with isoproterenol (+ISO, 30 min). The recordings for CFP and YFP were sampled at 10 sec intervals. The images shown are representative of a large array of images collected for this purpose.

When the AKAR2-AKAP12 cells are challenged with beta-adrenergic agonist, changes in image fluorescent density show a rapid, sustained decrease in the signal recorded from the CFP and a corresponding increase in the signal recorded for the YFP (citrine, Fig. 4), *i.e*., a FRET signal [11,12]. Signals recorded in the area indicated demonstrate that the biosensor-AKAP has been phosphorylated and that this phosphorylation yields a FRET signal from the "activated" AKAP. Thus, PKA-catalyzed phosphorylation of AKAP12, which is essential to its activation and function [14], yields a FRET signal from this biosensor. This is the first such image of an AKAP *"biosensed*" and recorded in the activated state, phosphorylated in response to the cellular stimulation with beta-adrenergic agonist.

**Figure 5:**
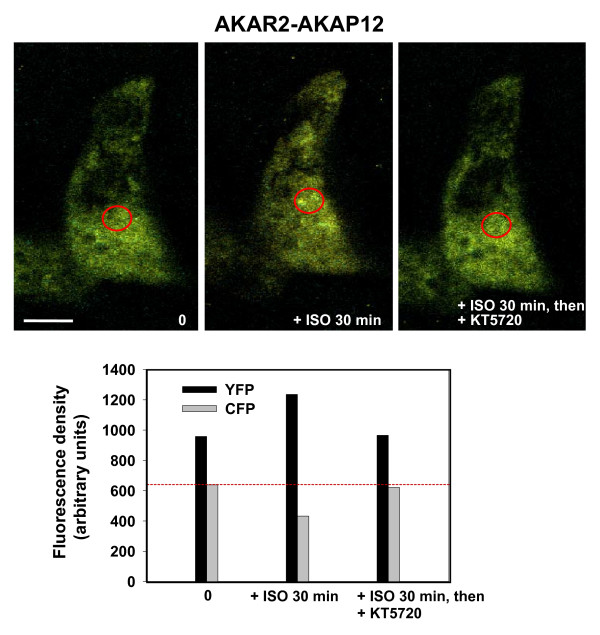
**FRET-based *"biosensing" *by AKAR2-AKAP12 in response to beta-adrenergic agonist: effects of addition of PKA inhibitor KT5720**. Stably transfected A431 clones expressing AKAR-AKAP12 were serum starved over night and then stimulated with beta-adrenergic agonist (isoproterenol, 10 μM) for 30 min. *Upper panel*, images were recorded for CFP and YFP in the absence (0), following the 30 min challenge with isoproterenol (+ISO, 30 min), and in the presence of isoproterenol for 30 min, then treated with PKA inhibitor KT5720 (+ISO 30 min, +KT5720) The measurements were made in the perinuclear, cytoplasmic area (open red circle) in which robust FRET signals from activated AKAR2-AKAP12 are observed. *Lower panel*, the fluorescence density measurements were collected from a large array of images collected for this purpose. The fluorescence density of CFP in the same area of the cells at basal state (-ISO) is highlighted by a red dashed line.

In order to probe the initial conclusion that the biosensor-AKAP12 was reporting the phosphorylated, "active" state necessary for its function in signaling, we employed several well-known tests as controls. If the FRET signal (increased YFP signal) reflected PKA-catalyzed phosphorylation of the AKAP12 biosensor, then we might expect that inhibition of PKA itself would impact the FRET signal. We treated cells with beta-adrenergic agonist for 30 min to stimulate the FRET signal and then treated the cells with the PKA inhibitor KT5720 that can be added to the cell cultures directly (Fig. 5). The FRET signal from the AKAP12 biosensor that increased in response to isoproterenol was attenuated sharply by the addition of KT5720. When KT5720 was added to the medium 5 min in advance of the isoproterenol, no FRET signal was recorded in response to the subsequent stimulation by isoproterenol (results not shown). Thus, chemical inhibition of PKA in live cells attenuates/blocks the ability of a beta-adrenergic agonist to stimulate the phosphorylation of AKAP12 biosensor as evidenced by the FRET signal.

**Figure 6:**
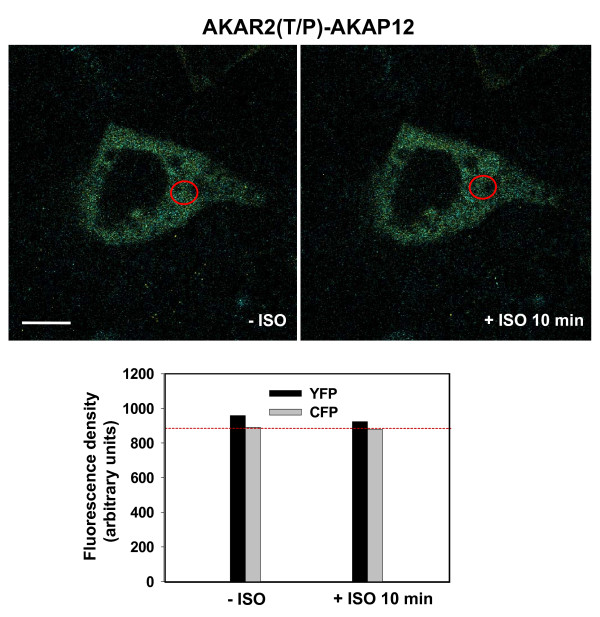
**FRET-based "*biosensing" *by AKAR2(T/P) mutant-AKAP12 in response to beta-adrenergic agonist**. Stably transfected A431 clones expressing AKAR2(T/P) mutant of AKAP12 (lacking the phosphorylation site for PKA in the substrate domain of the AKAR2 moiety) were serum starved over night and then untreated (-ISO) or treated with beta-adrenergic agonist (isoproterenol, 10 μM for 10 min (+ ISO 10 min). *Upper panel*, images and fluorescent densities recorded for CFP and YFP are displayed. The measurements were made in the perinuclear, cytoplasmic area (open red circle), an area in which robust FRET signals from activated AKAR2-AKAP12 are observed. *Lower panel*, the fluorescence density measurements were analyzed from a large array of images collected for this purpose. The fluorescence density of CFP in the same area of the cells at basal state (-ISO) is highlighted by a red dashed line.

Another test of the veracity of the biosensor to faithfully report on the phosphorylation of the AKAP12 fusion molecule by PKA, was to mutate the site of the PKA phosphorylation in the AKAR2 moiety, designated AKAR2(T/P)-AKAP12. The impact of this T-to-P substitution mutagenesis in the AKAR2 moiety of the AKAR2-AKAP12 biosensor on its ability to *"biosense*" was established. The levels of expression of the AKAR2(T/P)-AKAP12 in A431 cells was similar to that AKAR2-AKAP12 (results not shown). Treating cells for 10 (or 30 min, not shown) with beta-adrenergic agonist, resulted in *no *change in either the CFP or the YFP (FRET) signal in cells expressing the AKAR2(T/P)-AKAP12 mutant form of the biosensor (Fig. 6).

**Figure 7:**
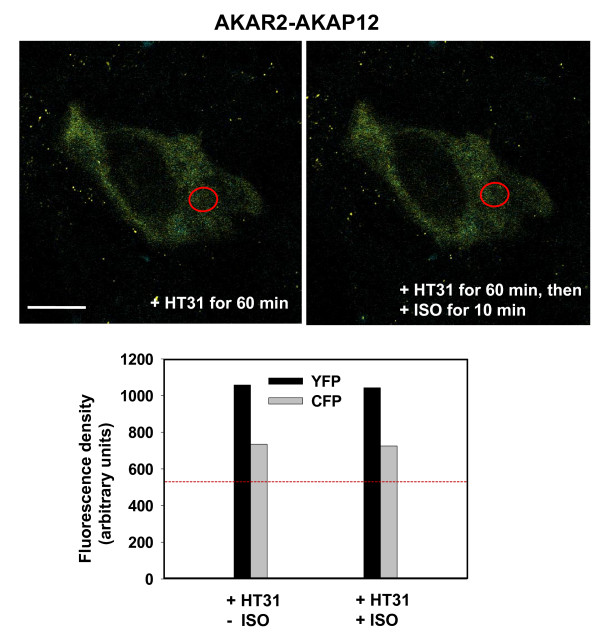
**FRET-based *"biosensing*" by AKAR2-AKAP12 in response to beta-adrenergic agonist: effects of HT-31 peptide interference of AKAP/RII subunit-binding**. Stably transfected A431 clones expressing AKAR-AKAP12 were serum starved over night, pre-treated with liposomes loaded with the HT-31 peptide (50 μM loading), and then stimulated with beta-adrenergic agonist (isoproterenol, 10 μM) for 10 min. *Upper panel*, images were recorded for CFP and YFP from the HT-31 peptide-treated cells that were then incubated either without isoproterenol (-ISO), or with isoproterenol (+ISO, 10 μM) for 10 min. The measurements were made in the perinuclear, cytoplasmic area (open red circle), an area in which robust FRET signals from activated AKAR2-AKAP12 are observed. *Lower panel*, the fluorescence density measurements were collected from a large array of images collected for this purpose. The fluorescence density of CFP in the control cells (-HT31) at basal state (-ISO) is highlighted by a red dashed line.

Finally, we tested the effects of the HT-31 peptide, which blocks PKA anchorage to AKAPs, on the ability of the AKAR2-AKAP12 to *"biosense*" phosphorylation of the biosensor AKAP by PKA. The HT-31 peptide was delivered in liposomes to A431 cells at 60 min prior to challenge of the cells with the beta-adrenergic agonist, isoproterenol. The results demonstrated that through blocking the interaction between the RII subunit of PKA and the AKAP, the AKAR2-AKAP12 no longer generates a FRET signal in response to isoproterenol-stimulated, PKA-catalyzed phosphorylation (Fig. 7). Next the cells were treated with the HT-31-prolyl mutant (HT-31p). This poly prolyl mutation of HT-31 peptide blocks the ability of the peptide to interfere with PKA RII subunit binding to AKAPs. Unlike the HT-31 peptide, the prolyl mutant no longer is able to block the FRET signal generated in response to stimulation with beta-adrenergic agonist (results not shown). The ability of HT-31 peptide, but not the HT-31p control peptide, to block AKAR2-AKAP12 *"biosensing*" agrees well with much earlier studies of the ability of the HT-31 peptide to block the RII subunit-binding domain/AKAP interaction essential to the dynamic localization of AKAP12 [17]. Likewise, mutation of the RII subunit-binding site of AKAP12 for PKA resulted in a mutant that could not rescue the functional responses of AKAP12 in cells made deficient of endogenous AKAP12 [14].

**Figure 8:**
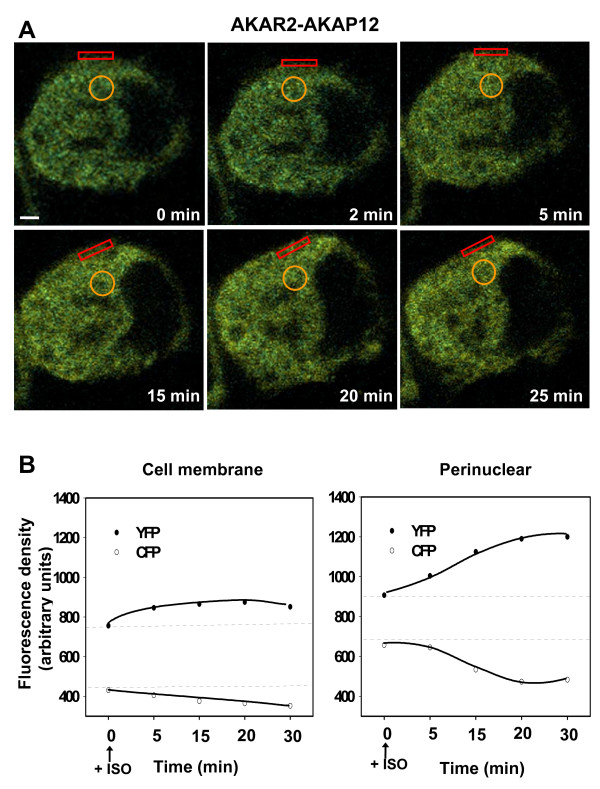
**FRET signaling from AKAR2-AKAP12 fusion protein "*biosenses*" dynamics of phosphorylated, activated AKAP**. *Panel A*, A431 cells were stably transfected with AKAR2-AKAP12. Cells were put in serum starvation for 12 hrs before the recording. Images were recorded for CFP and YFP in absence and presence of isoproterenol (10 μM) and sampled from time = "0" min until time = 30 min. The sampling of the FRET was confined to two cellular locales: the cell membrane (open red rectangles) and the perinuclear, cytoplasmic regions (open yellow circles). The images shown are representative of a large array of images collected for this purpose. *Panel B*, the fluorescence densities in cell membrane and perinuclear areas were recorded for CFP and for YFP in absence and presence of beta-adrenergic agonist. The data sets are from many samplings, performed on individual cells, and displayed as a representative fluorescence density scan over time.

We extended our analysis of AKAP12 phosphorylation dynamics by examining the spatial-temporal details of PKA-catalyzed phosphorylation of the scaffold, using the AKAR2-AKAP12 biosensor. Earlier it was reported that AKAP12 is found both localized to the cell membrane as well as throughout the cytoplasm in large punctate complexes [13-16]. In the unstimulated A431 cells, a modest AKAR2-AKAP12-derived FRET signal is observed, suggesting that at ambient levels of cyclic AMP and PKA activation, some cell membrane-associated, phosphorylated AKAR2-AKAP12 is present (Fig. 8A). For untreated A431 cells, the FRET signal images are similar to those of the eGFP-tagged AKAP12 [15]. AKAP5 and AKAP12 both display three PCDs that dictate increased proximity to the cell membrane in the absence of AKAP phosphorylation [5,6,16].

Stimulation of A431 cells with beta-adrenergic agonist provokes a 3-5 fold increase in the amount of AKAP12 localized to the cell membrane [16-18]. We analyzed the FRET signal from A431 cells expressing AKAR2-AKAP12 biosensor at various times following stimulation of the cells with beta-adrenergic agonist (Fig. 8A). Utilizing single cell recordings, FRET signals were scanned for two specific regions: a region which is largely perinuclear, cytoplasmic in character (open yellow circle) and a region that is largely cell membrane (open red, rectilinear box). Cells were treated with beta-adrenergic agonist and the FRET signal measured at various intervals over the next 30 min. Within the first 5 min of treatment with isoproterenol, FRET signals from the AKAP12 biosensor scanned at the cell membrane increased (Fig. 8B, left-handed panel). These data suggest that PKA-catalyzed phosphorylation, which is necessary for the receptor resensitization and receptor recycling functions of AKAP12, is readily *"biosensed*" at the cell membrane and reflects PKA-catalyzed phosphorylation of the scaffold which accumulates at the cell membrane during activation [13-15]. Over time, following stimulation by beta-adrenergic agonist, a steady-state appears to be reached in which the amount of AKAP12 phosphorylated at cell membrane remains rather constant, even though biochemical studies clearly demonstrate that it is accumulating first at the cell membrane and later in the cytoplasm where the mobile scaffold is associated with the internalized GPCR [4,13-15].

The FRET signal from the AKAR2-AKAP12 sampled from the perinuclear, cytoplasmic region of the cells (see open yellow circles) displays a forward, progressive increase to 30 min post-stimulation, the last time sampled (Fig. 8A and 8B, right-handed panel). The magnitude of the FRET signal over the 30 min period of stimulation of GPCR appears to reflect the progressive accumulation of AKAP12 scaffold at perinuclear, cytoplasmic regions noted earlier [13-15]. The scaffold, internalized and migrating in association with the internalized GPCR, clearly displays a FRET signal that is pronounced and progressively increasing over the 30 min of observation following treatment of the cells with isoproterenol. In the absence of AKAP12 phosphorylation (due to mutagenesis of the PKA RII subunit-binding site), desensitized GPCR have been shown to undergo internalization, but neither resensitization nor recycling [14]. During the interval of stimulation by beta-adrenergic agonist, AKAP12 becomes phosphorylated in response to activation of PKA and accumulates in the cytoplasm, where it catalyzes a necessary resensitization and recycling of desensitized beta_2_-adrenergic receptors.

## Conclusion

The current data shed important new light on the dynamics of AKAP12 in response to a challenge of cells with a beta-adrenergic agonist. It is the first time that a biosensor has been fused with a scaffold molecule in an attempt to yield a biosensor fusion protein designed to detect the phosphorylation and activation of a targeted PKA substrate, in this case AKAP12. The AKAR2-AKAP12 displays full ability to function in resensitization and recycling of GPCRs to the cell membrane, once they have undergone activation/desensitization. Although some details of the AKAP biology were revealed by static determinations and largely biochemical means [1-4], the ability to *"biosense*" only the AKAP12 molecules that undergo PKA-catalyzed phosphorylation and activation (with respect to function) in a time-space continuum was a worthwhile goal to seek. The biosensor fusion protein AKAR2-AKAP12 yielded a FRET signal upon stimulation of the cells with isoproterenol. The magnitude of the FRET signal suggests that only some of the AKAP12 at or in the vicinity of the cell membrane undergoes initial PKA-catalyzed phosphorylation. Stimulation of the GPCR results in a 3-5 fold increase in the cell membrane-associated AKAP12, some being physically-associated with the GPCR and some phosphorylated by PKA. The magnitude of the FRET signal would be most consistent with PKA-catalyzed phosphorylation of AKAP12 followed by eventual redistribution away from the membrane; this conclusion is buttressed by earlier immunohistochemical and biochemical analyses. We are able here to demonstrate the space-time localization of the phosphorylated, activated AKAPs (*i.e*., still phosphorylated). If the PKA-catalyzed phosphorylation occurred prior to beta-adrenergic agonist-induced accumulation of the AKAP at the membrane (which peaks within 5-10 min), then the increase in FRET would have been expected to be more robust.

The increase in the perinuclear, cytoplasmic FRET signal reflects the agonist-induced internalization of GPCR/phospho-AKAP12 complex, revealing for the first time those AKAR2-AKAP12 phosphorylated by PKA. The accumulation of the PKA-phosphorylated and activated form of the AKAP12 away from the cell membrane appears to be associated with the ability of AKAP12 to rescue desensitized GPCRs through a process of resensitization and eventual recycling to the cell membrane. The AKAR2-AKAP12 fusion protein is able to *"biosense*" the PKA-catalyzed phosphorylation of the scaffold and report on its cellular locale. The phosphorylation by PKA is necessary for the scaffold function in receptor resensitization and recycling, a cardinal process in the rescue of desensitized GPCRs, exemplified herein by study of beta_2_-adrenergic receptors. Clearly, the availability of an AKAP-biosensor that can report back on the phosphorylation status of AKAP with respect to PKA (and by suitable engineering, other interesting protein kinases, like Src [18]) permits the interrogation of the spatial-temporal sequence of events in which phosphorylated AKAP12 participates in the resensitization and recycling of GPCRs. This biosensor fusion protein strategy of use in study of AKAP biology will likely provide powerful new tools for the analysis of the space-time continuum for other dynamic, multivalent scaffolds such as the Dishevelleds [19].

## Methods

### Cell culture

Human epidermoid carcinoma cells (A431) were maintained in Dulbecco's modified Eagle's medium supplemented with 10% fetal bovine serum (HyClone, Logan, UT), penicillin (60 μg/ml), and streptomycin (100 μg/ml) and grown in a humidified atmosphere of 5% CO2 and 95% air at 37°C. A431 cells were transfected with AKAR2 pcDNA3.1 or AKAR2-AKAP12 pcDNA3 *hygro *using Lipofectinamine Plus™ and Plus (Invitrogen) according to the manufacturer's protocol, and viable clones were selected in 400 μg/ml of the neomycin analogue G418 or hygromycin.

### Construction of AKAR2-AKAP12

AKAR2 was amplified by using PCR with primers containing Nhe I and Kpn1 at N or C terminal. AKAP2 was inserted in PcDNA3 *hygro *between Nhe I and Kpn 1. AKAP12' (1-2180) was amplified by using PCR with primers containing Kpn1 and BamH1 at H_2_N-terminus or -COOH-terminus. The PCR product then was subcloned in the AKAR2-pcDNA3 *hygro*. AKAP12" (2181-5346) was amplified by using PCR with primers containing BamH1 and Not1 at H_2_N-terminus or -COOH-terminus. This PCR product then was subcloned into the pcDNA3-AKAR2-AKAP'construct to form a complete AKAR2-AKAP12 pcDNA3 *hygro *construct. Sequences of all primers used are available from the authors upon request. Threonine-to-Proline substitution mutant of AKAR2-AKAP12 at PKA phosphorylation site in AKAR2 was engineered by standard protocol for PCR-mediated mutagenesis. All the PCR was performed using Pfu polymerase (Stratagene). The integrity of the amplified sequence was confirmed by DNA sequencing.

### Immunoblotting

A431 cell extracts were probed for AKAP5 or AKAP12 expression by immunoblotting. Cells were stimulated with 10 μM isoproterenol for different time intervals. Cells were harvested and lysed in a lysis buffer (1% Triton X-100, 0.5% Nonidet-40, 10 mM dithiothreitol, 5 μg/ml aprotinin, 5 μg/ml leupeptin, 100 μg/ml bacitracin, 100 μg/ml benzamidine, 2 mM sodium orthovanadate, 150 mM NaCl, 5 mM EDTA, 50 mM NaF, 40 mM sodium pyrophosphate, 50 mM KH_2_PO_4_, 10 mM sodium molybdate, and 20 mM Tris-HCl, pH 7.4) at 4°C for 20 min

### Desensitization and resensitization of β2AR

Two days prior to the analysis of agonist-induced desensitization, the A431 cells were seeded in 96-well microtiter plates at a density of 25,000-50,000 cells/well. Routinely cells were serum-starved overnight, prior to analysis. Desensitization was accomplished by pretreating the cells with the beta-adrenergic agonist isoproterenol (10 μM) for 30 min. Under these conditions, subsequent beta-adrenergic stimulation of cyclic AMP accumulation is severely blunted and the number of cell-surface receptors declines precipitously as the receptors are sequestered and internalized. Details of the desensitization protocol are described elsewhere [14-17].

### Knockdown of AKAP12 with antisense morpholinos

Antisense morpholino oligonucleotides (morpholinos) were designed, synthesized, and purified to cell culture grade (Gene Tools, LLC). The morpholinos and protocol for knockdown (KD) of endogenous AKAP12 expression were optimized and reported earlier [18]. The extent of the suppression of gravin expression by antisense morpholinos in these studies was >85%. The antisense morpholinos were designed to target the 5'-untranslated region of the mRNA for native AKAP12 and do not recognize AKAP12 mRNA transcribed from the expression vector (pcDNA3). Prior to their addition to A431 cell cultures, morpholinos were mixed in a ratio of 1:1 (w/w) with EPEI special delivery solution (Gene Tools, LLC). Cells were treated with the anti-AKAP12 morpholinos (5 μg/ml) for 3 days. Whole cell lysates of the morpholino-treated cells were subjected to SDS-PAGE and the resolved proteins were blotted and stained with anti-gravin antibody. Under standard conditions, morpholinos antisense for AKAP12 suppressed the cellular level of the AKAP by more than 90% [13,18]. An additional treatment with morpholinos antisense to AKAP12 was performed prior to transient transfection of the cells with either wild-type (WT) or mutant forms of AKAP12. The morpholinos are designed to suppress the expression of endogenous AKAP12 at a 5'-untranslated region, not interfering with the AKAP12 (or AKAR2-AKAP12) expressed through use of mammalian expression vector, pcDNA3. Following this protocol, cells were analyzed for beta-adrenergic agonist-induced (*i.e*. Iso, 10 μM) desensitization, as well as the recovery (*i.e*. resensitization and recycling of beta_2_-adrenergic receptors) after washout of agonist.

### Confocal fluorescence and fluorescence emission

Measurements were performed at 37°C on Zeiss LSM 510 scanning laser confocal microscope with 40×/1.3 numerical aperture Plan Apochromatic oil immersion objective lens with three laser lines (targeting specifically CFP, YFP (citrine variant), and DAPI). The fluorescence was detected by 3 high efficiency photomultipliers (CFP, 473-495 nm; YFP and FRET, 527-591 nm; DAPI, 435-485). Cells were scanned at 400 lines/s and with four times averaging. Images (512 × 512 pixels) were optimized and overlay images (YFP/CFP or YFP/CFP/DAPI) created with LSM image browser. A time series consisting of images that were captured every 5 min was obtained for each cell. Cells were stimulated with beta-adrenergic agonist (isoproterenol) in either the presence or the absence of indicated chemical inhibitors of enzymes, as described above.

## Abbreviations

AKAP: A-kinase anchoring protein; AKAP5: AKAP79; AKAP12: gravin, AKAP250, SSECKS; A-kinase, PKA: protein kinase A; CFP: cyan fluorescent protein; DAPI: 4',6-diamidino-2-phenylindole; GFP: green fluorescent protein; GPCR: G protein-coupled receptor; SDS-PAGE: sodium dodecyl sulfate-polyacrylamide gel electrophoresis; YFP: yellow fluorescent protein, in these studies the variant citrine.

## Competing interests

The authors declare that they have no competing interests.

## Authors' contributions

JT designed and constructed the biosensor fusion protein, performed many of the experiments, gathered the data, and outlined the contents of the manuscript. HYW performed some of the experiments, collaborated on project from design to completion, and contributed to the editing of the manuscript. CCM participated in the evolution of the overarching study design, in the interrogation of the experimental design during all phases, and drafted/edited the final manuscript. All authors read and approved the final manuscript.
